# HMBG1 as a Driver of Inflammatory and Immune Processes in the Pathogenesis of Ocular Diseases

**DOI:** 10.1155/2018/5195290

**Published:** 2018-10-24

**Authors:** Yi Liu, Guo-Bin Zhuang, Xue-Zhi Zhou

**Affiliations:** ^1^Department of Ophthalmology, Nanjing Hospital of Chinese Medicine, The Third Affiliated Hospital of Nanjing University of Chinese Medicine, Nanjing, Jiangsu 210001, China; ^2^Department of Ophthalmology, Quanzhou First Hospital, Quanzhou, Fujian 362000, China; ^3^Department of Ophthalmology, Xiangya Hospital, Central South University, Changsha, Hunan 410078, China

## Abstract

High-mobility group box 1 (HMGB1) is a nuclear protein that can also act as an extracellular trigger of inflammation, proliferation, and migration in eye diseases. It induces signaling pathways by binding to the receptor for advanced glycation end products (RAGE) and Toll-like receptors (TLRs) 2, 4, and 9. This proinflammatory activity is considered to be important in the pathogenesis of a wide range of ocular diseases resulting from hemodynamic changes, presence of neovascular endothelial cells, secretion of intraocular immune factors or inflammation, and apoptosis of retinal cell layers. Further work is needed to elucidate in detail how HMGB1 contributes to ocular disease and how its damaging activity can be modulated. In this review, we summarize current knowledge on HMGB1 as a ligand that can evoke inflammation and immune responses in ocular diseases.

## 1. Introduction

The eye is a relatively independent and closed system in which various tissues and cells interact in complex ways [1]. Many eye diseases have been described, such as glaucoma, keratitis, diabetic retinopathy, and age-related macular degeneration, and these diseases often arise as a result of pathogenic immune responses against eye autoantigens or as a local manifestation of a systemic autoimmune response. Although development of new drugs and innovations in surgical methods has led to effective treatment of ocular diseases, how these diseases occur and progress remains poorly understood. Several studies indicate that ocular diseases have a close relationship with autoimmune reactions and inflammatory responses [2–5], and a key protein in such processes is high-mobility group box 1 (HMGB1) [6–8]. The present review examines the role of HMGB1 in eye diseases.

HMGB1 is a DNA-binding nuclear protein discovered over 30 years ago [9–11]. Normally, this protein exists in the cell nucleus; when it is released outside the cell, it becomes an immune-inflammatory factor [12–14]. It can be released from damaged cells or secreted by activated immune cells such as macrophages, dendritic cells (DCs), and natural killer cells [15–18]. It may induce signaling pathways by binding to the receptor for advanced glycation end products (RAGE) and Toll-like receptors (TLRs) 2, 4, and 9 [19, 20].

HMGB1 is closely related to many inflammatory diseases, such as ischemia of liver and kidney, hepatitis, arthritis, stroke, ischemia of liver and kidney, sepsis, and systemic lupus erythematosus [21–25]. In this review, our focus is the important role of HMGB1 in inflammatory immune eye diseases, including keratitis, uveitis, dry eye, diabetic retinopathy, and retinal degeneration. Inhibition of this protein may be an effective new treatment for patients with immune-inflammatory eye disease. We suggest that more research with both animal and human models is needed to confirm that HMGB1 has the therapeutic potential suggested by initial studies.

## 2. HMGB1 Structure

HMGB1 was identified as a nonhistone chromatin-binding protein about 30 years ago [9–11]. This protein is abundantly expressed in nearly all eukaryotic cells [26]. It acts as a damage-associated molecular pattern (DAMP) molecule [27]. HMGB1 is a member of the high-mobility group (HMG) chromosomal protein family [28]. HMG chromosomal proteins are divided into the three superfamilies HMGB, HMGN, and HMGA [29]. Human HMGB1 is an alarmin encoded by a single gene that is located on chromosome 13q12 [30]. In most cell types, HMGB1 is mainly located in the nucleus under physiological conditions. When cells are stimulated or suffer injury or death, HMGB1 translocates from inside to outside the cell [31, 32]. HMGB1 contains 215 amino acids arranged into two DNA-binding domains (HMG A box and HMG B box) and one C-terminal acidic tail, which contains a stretch of approximately 30 glutamic and aspartic acid residues [27, 33]. The HMG A and B boxes can bind to DNA and participate in DNA folding and twisting [34–36]. The HMG B box causes macrophages to secrete additional proinflammatory cytokines; in fact, the B box on its own can trigger the same processes as full-length HMGB1 [27, 37]. The A box can antagonize cytokine activity [38, 39]. The spatial arrangement of A and B boxes was regulated by C-terminal acidic tail. [10, 40, 41].

Each HMGB1 domain interacts with different receptors, and these interactions regulate the biological activity of extracellar HMGB1. TLR4 is binding with residues 89–108 of HMGB1 [42, 43], while RAGE is binding with residues 150–184 of HMGB1 [44]. Within the C-terminal acidic tail (residues 186–215), residues 201–205 exert anti-inflammatory activity [45]. Two nuclear localization sequences in HMGB1 may stabilize the chromatin structure and regulate gene transcription.

## 3. Expression of HMGB1 in Eye Disease

Numerous studies suggest that HMGB1 may contribute to eye disease by acting as an inflammatory cytokine. In a model of retinal ischemia reperfusion injury and inflammation, HMGB1 expression is upregulated in retinal pigment epithelium, retinal endothelial cells, ganglion cells, Müller cells, astrocytes, and photoreceptors [46]. Chronic epithelial cell damage to the cornea or lacrimal glands triggers HMGB1 secretion, which initiates an inflammatory cycle [47]. HMGB1 levels are elevated in the cytoplasm and extracellular space in eye disease. All these studies indicate that HMGB1 is abundantly expressed in ocular diseases, which reflects its role in contributing to these disorders.

## 4. HMGB1 and Diabetic Retinopathy

Dysfunction of retinal endothelial cells, damage to the blood-retinal barrier, ischemia, and retinal neovascularization are the characters of diabetic retinopathy at different stages [48]. Inflammation factor-mediated damage to the retina and optic nerve are key factors in the pathogenesis of diabetic retinopathy, which appears to involve continuous, low-level inflammation [49]. HMGB1 helps drive this process by acting as a late inflammatory factor [50].

In patients with proliferative diabetic retinopathy (PDR), HMGB1 levels in vitreous humor increased significantly [51]. The expression level of HMGB1 in vitreous body of patients with active PDR is significantly higher than that of inactive PDR patients [52]. HMGB1 may interact with vascular adhesion protein 1 (VAP-1), 8-hydroxy-2'-deoxyguanosine (8-OHdG), and heme oxygenase-1 (HO-1) in contributing to the pathogenesis of inflammation and angiogenesis in PDR [53].

HMGB1 is involved in the pathogenesis of diabetic retinopathy through the combination of RAGE and TLR9 [53]. In BV2 cells, a signaling pathway mediated by HMGB1, RAGE, TLR4, p38, and nuclear factor kappa light chain enhancer of activated B cells (NF-κB) activates matrix metalloprotease-9 in response to high glucose levels [54]. In RGC-5 cells, short interfering RNA against HMGB1 obviously downregulates expression of TLR4 and NF-κB mRNA in response to high glucose [55]. Treating human retinal endothelial cells with high glucose activates HMGB1 to trigger signaling by ERK1/2 and NF-κB, which in turn induces inflammatory responses and break vascular barrier of retinal [56].

The presence of diabetes in rats or intravitreal injection of HMGB1 in normal rats significantly upregulates the levels of mRNAs encoding SDF-1/CXCL12 receptor CXCR4, as well as levels of HIF-1, EGR-1, TK-2, and CXCL12/CXCR4 proteins in the chemokine axis [57]. In diabetic retinopathy, HMGB1 is closely related not only to the apoptosis of nerve cells but also to neovascularization; the latter involves STAT-3 as a downstream effector of HMGB1 to drive angiogenesis [58]. In human retinal microvascular endothelial cells, HMGB1 significantly upregulates levels of IL-1*β* and reactive oxygen species, as well as expression of NADPH oxidase 2 (Nox2), caspase-3, and PARP-1 [59]. HMGB1 exerts these proinflammatory and angiogenic effects via TNFα and VEGF [57].

**Figure 1:**
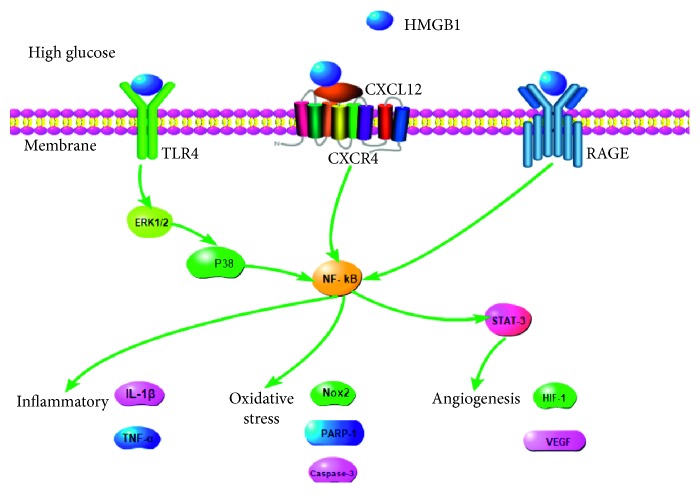
HMGB1 signal by binding to TLR4, CXCR4, and RAGE to moderate BV2 cells, RGC cells, retinal endothelial cells, and retinal microvascular endothelial cells function in PDR. In these cells, HMGB1 binds TLR4 and activates ERK1/2-P38-NF-*κ*B signal pathway. HMGB1 also binds RAGE and upregulated the expression of NF-*κ*B. In human retinal microvascular endothelial cells, HMGB1 binds CXCR4 to activate NF-*κ*B/STAT-3 signal pathway. These three signaling has been implicated in cell inflammatory, oxidative stress, and angiogenesis.

Together, these studies demonstrate that HMGB1 helps to drive diabetic retinopathy in two ways. HMGB1 binds to TLR4 and RAGE to trigger nerve cell death. At the same time, HMGB1 triggers release of proangiogenic factors to induce pathological neovascularization (Figure 1).

## 5. HMGB1 and Uveitis

Inflammation of the iris, ciliary body, vitreous body, retina, or choroid is a typical feature of uveitis. It refers to a group of intraocular inflammatory diseases that arise without a clear infectious trigger and is a leading cause of visual impairment and legal blindness, particularly in the younger population. It contributes to 10–15% of all cases of blindness (defined as central visual acuity of 1/10 or less in the better eye) in developed countries. Such blindness arises mainly as a result of macular edema, ocular hypertonia, or retinal ischemia.

Topical and systemic anti-inflammatory and immunosuppressive medications can completely eliminate inflammatory signs such as keratic precipitates, aqueous flare, miosis, cortical (equatorial) cataracts, vitreal opacification, and lesions of the fundus or optic nerve head. Inflammation can recur after such therapy.

Uveitis is closely related to the immune response associated with T cells. Indeed, experimental autoimmune uveitis is induced by autogenous reactive T-cell injection in rodents [60].

In models of experimental autoimmune uveoretinitis, RAGE is upregulated in inflammatory cells, including macrophages infiltrating the anterior chamber, vitreous cavity, retina, subretinal space, and choroid [61]. HMGB1 activates RAGE, promoting the release of TNF-*α*, ICAM-1, VCAM-1, and IL-6. HMGB1 also induces CXCL12 release by infiltrating effector T cells [62]. Release of intraocular HMGB1 and CXCL12 at one day after cell transfer may attract inflammatory T cells [63]. Elevated HMGB1 levels may subsequently be associated with the participation of T cells and the retina lesion [62]. Autoimmune uveitis is activated by IRBP-specific T cells [64]. These kinds of T cells were interacted with substantive cells including Müller cells, DCs, astrocytes, and microglia. These cells produce HMGB1 via Fas/FasL-mediated signaling. RIP2 is also involved in HMGB1 release which is deeply related with Fas in uveitis [65]. This suggests the active involvement of the HMGB1-Fas-RIP2 axis in uveitis.

**Figure 2:**
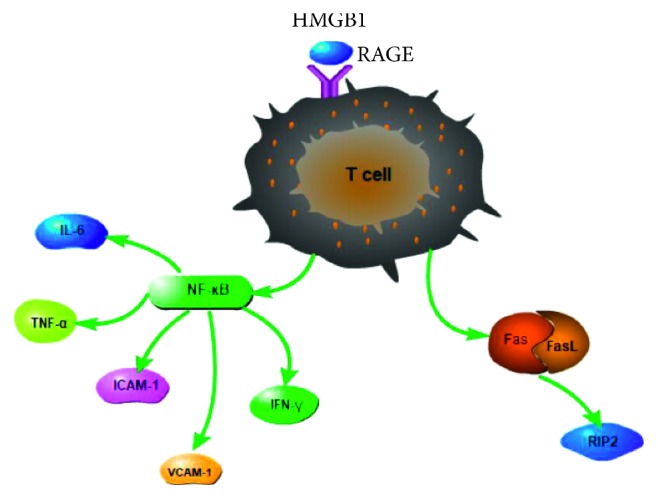
HMGB1 signals by binding to RAGE to activate T-cell cytokine release and Fas/FasL signal pathway in uveitis. In uveoretinitis, HMGB1 binds RAGE and activates NF-*κ*B, promoting the release of TNF-*α*, IL-6, ICAM-1, and VCAM-1. HMGB1 also can activate Fas/FasL-mediated signaling. In Behcet's disease, expression of proinflammatory cytokines such as TNF-*α*, IL-6, and IFN-*γ* in neutrophils is enhanced by extracellular HMGB1, which acts via the NF-*κ*B pathway.

HMGB1 plays an important role in Behcet's disease, one of the forms of uveitis. Although this disease involves elevated HMGB1 levels in serum, these serum levels do not appear to be associated with disease activity or specific organ damage [66]. Neutrophils help drive Behcet's disease, and expression of proinflammatory cytokines such as TNF-*α*, IFN-*γ*, and IL-6 in neutrophils is enhanced by extracellular HMGB1, which acts via the NF-*κ*B pathway [6]. These findings suggest that pathogenesis of Behcet's disease is linked to the ability of HMGB1 to activate the NF-*κ*B signaling pathway and thereby stimulate the secretion of inflammatory cytokines (Figure 2).

## 6. HMGB1 and AMD

AMD is a disease of macular degeneration, which is the main cause of irreversible central vision loss in the elderly in developed countries [67]. This vision loss is thought to involve degeneration of retinal pigment epithelial cells and the overlying photoreceptors, which rely on the retinal pigment epithelium for trophic support.

In a culture model of AMD in which retinal pigment epithelial cells are treated with NaIO_3_, HMGB1 is released from the nucleus [68]. HMGB1 is also released from the nucleus and secreted by retinal pigment epithelial cells in response to oxidative stress [69]. Depleting HMGB1 from the culture medium inhibits the induction of TNF-*α* production by retinal pigment epithelial cells [70]. Treating wild-type retinal pigment epithelial cells with poly(I:C) and Z-VAD induces the secretion of substantial amounts of HMGB1, which is completely blocked by deleting the *Rip3* gene from the epithelial cells [70]. The release of HMGB1 from necrotic retinal pigment epithelial cells can increase the generation of IL-6 and TNF-*α* in macrophages and release of these cytokines from retinal pigment epithelial cells; the cytokine release is inhibited by deleting the *Rip3* gene [71]. In another culture model of AMD in which retinal pigment epithelial cells are treated with poly(I:C) and Z-VAD, NF-*κ*B signaling is directly modulated by HMGB1 and receptor-interacting protein kinase 3 (Rip3) [72].

## 7. HMGB1 and Glaucoma

Acute glaucoma is an ophthalmic emergency that progresses to blindness if untreated. Obstruction of the drainage of the aqueous humour as a result of narrowing or closure of the anterior chamber angle causes high intraocular pressure, which damages the optic nerve and triggers retinal ischemic reperfusion injury and retinal ganglion cell death. In rats, HMGB1 has been linked to glaucoma induced by elevated intraocular pressure [73]. Treating cocultures of retinal ganglion cells and glia with HMGB1 causes ganglion cell death [74].

HMGB1 significantly upregulates cleaved caspase-8 and activates the canonical NLRP3-inflammasome, which induces the processing of IL-1*β* via pathways dependent on caspase-1 and caspase-8 and leads to retinal ganglion cell death and retinal damage [75]. In a model of retinal IR, HMGB1 appears to upregulate expression of the p65 subunit of p-NF-*κ*B and activate the NF-*κ*B pathway [75]. RAGE and TLR4 mediate the ability of HMGB1 to activate proinflammatory pathways and enhance retinal damage. Treating ganglion cell layer neurons with anti-HMGB1 antibody improves their survival more than knocking out either RAGE or TLR4 expression [73].

## 8. HMGB1 and Corneal Diseases

One of the most general reasons of microbial keratitis is *pseudomonas aeruginosa* which is widespread in patients with prolonged contact lenses and impaired immunity. Impaired healing of corneal wounds resulting from alkaline burn is also a major cause of impaired vision, and strategies for treatment should be improved in order to avoid keratoplasty. These two kinds of corneal diseases are refractory: so far, there is no effective treatment to cure them. HMGB1 is involved in the pathogenesis of both diseases, and it may have potential as a therapeutic target [76].

In *P. aeruginosa*-induced keratitis, elevated levels of extracellular HMGB1 exacerbate inflammation by stimulating neutrophils via RAGE and TLR pathways [77]. Attenuating HMGB1-mediated disease progression can help treat keratitis. In an animal model of bacterial keratitis, silencing HMBG1 reduces expression of IL-1*β*, MIP-2, and TNF-*α* by inhibition of NF-*κ*B as well as the receptors TLR4 and RAGE [78]. Silencing HMGB1 also downregulates CXCL12 and CXCR4 expression. Silencing HMGB1 in a mouse model of *P. aeruginosa*-induced keratitis increases levels of anti-inflammatory cytokines, reduces levels of inflammatory cytokines and neutrophil infiltration, and improves prognosis [79].

**Figure 3:**
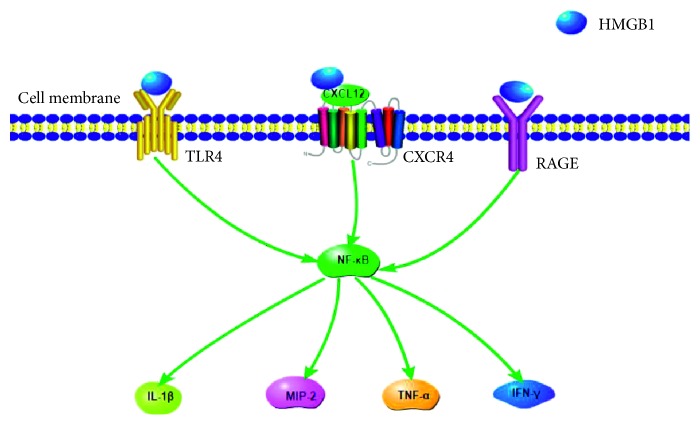
Signaling pathways downstream of RAGE, TLR4, and CXCR4 mediate the effects of HMGB1 in corneal diseases. RAGE signals, TLR4 signals, and CXCR4 signals promote the activation of NF-*κ*B. After the activation of NF-*κ*B, the inflammatory cytokines such as IL-1*β*, MIP-2, TNF-*α*, and IFN-*γ* are released.

HMGB1 also plays an important role in corneal healing and corneal neovascularization. There are two neovascularization processes. One of these processes is the production of endothelial cells from existing blood vessels. The other process is initiated by bone-derived progenitor cells, which then drive endothelial progenitor cells to target sites and into the vascular endothelium. Endothelial cells are also bone-derived progenitor cells. Then, the progenitor cells differentiate into endothelial cells and support the construction of new blood vessels. HMGB1 accelerates corneal neovascularization via TLR4- and RAGE-mediated signaling pathways by activating macrophages, stimulating the secretion of proangiogenic factors and promoting TLR4-induced recruitment of endothelial progenitor cells [80]. Of the three HMBG1 receptors (TLR2, TLR4, and RAGE), HMBG1 appears to act via TLR4 to drive corneal neovascularization. Nuclear HMGB1 interacts with TLR2, which is present in human and rat corneal epithelial cells, but this receptor has no effect on secreted HMGB1 [81]. Silencing TLR2 does not significantly affect corneal neovascularization [81]. Alkaline burns upregulating RAGE and TLR4 in mouse cornea with similar kinetics, but the upregulation of RAGE is small [47] (Figure 3).

## 9. Conclusion and Perspectives

Although tremendous advances have been made in the study of the structure, release patterns, and receptors of HMGB1, the intracellular signaling pathways that it modulates are still poorly understood, especially its interaction with TLRs and RAGE. This is important to understand in order to clarify how HMGB1 contributes to ocular diseases and develop better therapeutic strategies. HMGB1 antagonists have shown significant therapeutic effects in animal models, but more extensive trials are needed in animals and ultimately in humans. Future studies should also address whether posttranslational modifications affect HMGB1-mediated signaling and whether other carrier or binding proteins assist HMGB1 in modulating cytokine activity in ocular diseases. This work may solidify HMGB1 as a novel therapeutic approach.
